# A freely accessible, evidence based, objective system of analysis of posterior capsular opacification ; Evidence for its validity and reliability

**DOI:** 10.1186/1471-2415-5-9

**Published:** 2005-04-07

**Authors:** Tariq M Aslam, Niall Patton, Jim Graham

**Affiliations:** 1 Manchester Eye Hospital, UK; 2 Sir Charles Gairdner Hospital, Perth, Australia; 3 Dept. Imaging Sciences and Biomedical Engineering, Manchester University, UK

## Abstract

**Background:**

The aim of this study was to develop a system of computerised analysis of digital images of posterior capsule opacification (PCO) that is evidence based, objective and freely available. The paper will present evidence for the reliability and validity of the developed system.

**Methods:**

The system of PCO analysis was developed considering current published evidence on visual significance of PCO and additional investigative analysis of PCO images. Details of the image processing and analysis steps are discussed and a final system that measures an entropy score weighted toward proximity to central areas is described. In order to assess validity, the systems ability to measure PCO progression is assessed along with the visual significance of its final computerised scores. Reliability of the system is also assessed.

**Results:**

The final system runs successfully and is simple to use. Analyses of PCO by the system show an ability to detect early progression of PCO as well as detection of visually significant PCO. Images with no clinical PCO produce very low scores in the analysis. Reliability of the system of analysis is shown to be satisfactory.

**Conclusion:**

This paper presents a system of PCO analysis that is evidence based, objective and clinically useful. Substantial evidence is provided for its validity and reliability.

## Background

Despite advances in the practice of cataract surgery and intraocular lens implantation, posterior capsule opacification remains the most common post-operative cause of morbidity[1]. However, there is currently no consensus on an optimal PCO quantification method. The main objective systems of analysis, POCO[2] and AQUA[3] systems are not freely available. They do not incorporate whether PCO is peripheral or central into calculations and show limited evidence for validity. The EPCO system [4] has been assessed for evidence of construct validity [5] but is still subjective. The POCO system[6] is also subjective and is not convincing for analysis of PCO in terms of measuring progression or visual significance. There is clearly a need for a universally acceptable measure of PCO [7] that would be objective enough for scientific analysis and yet not exclude the majority of researchers by its lack of free availability. It should be based upon current evidence on the visual significance of PCO and be tested to be both valid and reliable. This paper presents the first such system of analysis.

A principal problem in any system of analysis of digital images of PCO is that of artefactual light reflections spoiling the image. Although resilient to such artefactual corruption, the presented system is designed for use on images that have had some prior mechanism of reducing unwanted reflections. [4,7] For this study all testing and development was done on images created to be free of reflections by merging of two or more original images using commercially available and common software. [5] This step has been previously demonstrated as valid and reliable [5]

The presented program is not proposed as the ultimate measurement system for PCO. We would expect to continuously update the system with future research findings as well as data on analyses. These data may be used to refine certain parameters and factors used in calculations and algorithms.

The aim of this study was to develop a system of computerised analysis of digital images of PCO that is evidence based and objective. Evidence for validity and reliability of the final system will be presented.

## Methods

### Development of software

This system was designed and programmed using the MatLab programming platform(MatLabTM, Matrix Laboratory,^© ^The Mathworks, inc, MA). All programming design and writing of code was by T M Aslam and for clarity the system is hence referred to as the Aslam analysis (AA) in this paper. All images used in development of the system were of a different subset to those used in assessment of validity and reliability of the final analysis system. Informed consent was obtained from all patients involved and the study was approved by local ethics committee, in accordance with declaration of Helsinki.

**Figure 1:**
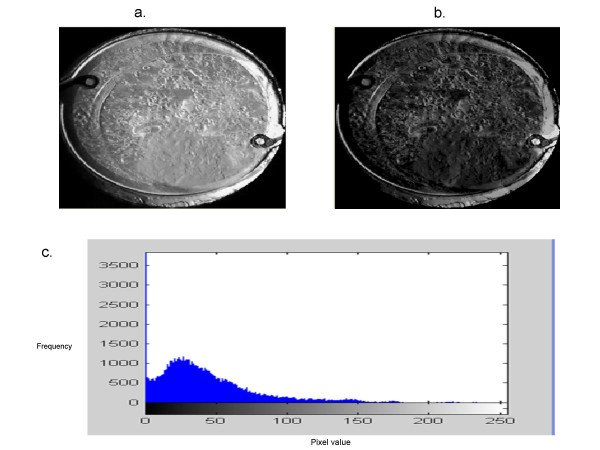
a. Original greyscale image, b. background removed, c. image histogram

**Figure 2:**
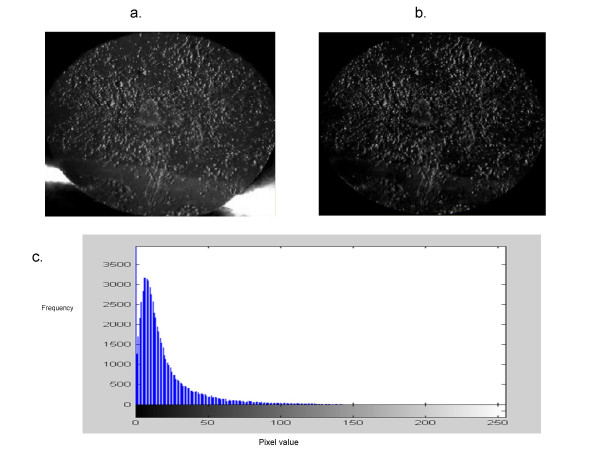
a. Original greyscale image, b. background removed, c. image histogram

The first problem encountered was one of unequal illumination in the images. Even with the large areas of aberrant light reflections removed, using merging with similar but unspoilt images, a generally variable background illumination of the entire image may cause errors in image analysis. This is solved in the AA system with an image processing step of background illumination removal. This is achieved through image manipulation techniques known as morphological opening[8] to estimate the background illumination. The resulting foreground PCO is freed of illumination variations. (fig 1b, 2b)

Next, unwanted outer segments of images, that include, for example, iris segments, must be excluded from the analysis. The desired area of interest involving the optic of the lens or area within the pupil is isolated in the AA system by the user marking out the corners of the region of interest. These primary steps lead to an isolated region of interest that is free from variations in illumination and ready for analysis. For this study, the central 3.5 mm of posterior capsule, centred using pupil borders, was used for all analyses. The dimensions of this area was noted in terms of pixel size using known diameter of intra-ocular optic in the images as a guide. This segment of PCO has been previously confirmed as being visually significant. [5]

It is established that areas of PCO distant from the centre of the visual axis have a reduced effect on vision compared to areas at the centre.[5] Although the exact mathematical relationship of this is unknown the AA system incorporates the principle of this research finding in its analysis. To do this the region to be analysed was split into multiple smaller subunits and each unit considered separately for analysis. Modifications to the analysis results of each subunit were then made depending on the distance of that subunit from the central visual axis. The further a unit was from central visual axis, the more the value of its texture would be attenuated. Finally texture scores for all subunits is summed to give a final total score. The factor used to modify PCO score according to distance needed to be large enough to exert the required effect of weighting scores towards the centre of the image, but not so large as to render the system insensitive to small amounts of peripheral PCO. Similarly, subunit size was adjusted until it was found to be large enough to incorporate significant PCO textural objects but small enough for a satisfactory total number of subunit regions to be analysed to allow for central weighting mechanism to function. Various combinations of subregion size and image score weighting were trialled before a compromise was achieved. This involved dividing each image into 121 total subunits.

Development of the PCO score itself involved considering many possible image processing and analytical techniques. Current evidence on PCO suggests that its texture, determined from retro-illuminated digital images, could be used to determine its visual significance.[2,9] Histogram analysis[10] suggested that texture analysis of the PCO might be feasible using statistical properties of the intensity histogram.(Fig 1c, 2c) Intensity levels of trial images with significant PCO showed distribution with distinct peaks at varying levels of intensity. The histograms all showed low dynamic range with narrow width compared to the entire grey scale. During development of the system, various descriptors of texture based on the intensity histogram of a region were calculated for potential use in analysis of PCO images of patients.[10] Entropy was found to provide the most useful form of texture measure. This corresponds with the fact that entropy tends to be higher in coarser textures, such as is found in pearl type opacification, that is proposed to be especially visually significant.[11] Indeed, such pearl type opacification areas showed more irregular and random peaks in histograms studied. In investigations detailed below, only the entropy measure showed consistent features of validity, and this measure was chosen as the most suitable marker for the analysis system.

Even after the above processing steps, images with no visible PCO and in which PCO would not be expected still showed on histogram analysis to have image areas with low but significant intensities of up to 15 units of brightness that contributed to elevated entropy scores. This was due to variations in intensity unrelated to significant PCO such as minimal persistent reflections, lens imperfections, photographic noise [12] and thin films of material developed over the lens. When the AA system was programmed to except this subset of values it appeared to have much better face validity for analysing small or no amounts of PCO, whilst maintaining face validity for analysis of large amounts of PCO in patients attending for capsulotomy.

Although there was apparent face validity and content validity of the final developed system, it was submitted for further testing before any confidence was held in results of its analyses. Experiments that provide evidence for such confidence in the validity and reliability of the AA system are now discussed.

### Testing for validity

#### Measurement of progression by AA system

In order to test the system's ability to measure progression, digital images of 12 patients within a month of cataract extraction were analysed along with images taken of the same patients of 12–18 months after cataract surgery. On inspection of these images many of the patients had evidently undergone PCO progression from no or nearly no PCO to visible but mainly peripheral PCO. One would expect a model system to produce values reflecting very low levels of PCO in patients within a month of surgery, and for those values to be increased by 18 months after cataract extraction. The results for the AA system were graphically plotted and paired t-tests done to assess whether findings were significantly different in the two groups.

#### Visual relevance of AA analysis

Evidence for both construct validity (agreement with theoretical expectations) and convergence validity (relation to associated factors) came from a study involving thirty patients (33 eyes) that were recruited after having been referred for potential Nd: YAG capsulotomy.

On attendance, patients had CS vision tested with Pelli-Robson charts. Patients were dilated and the posterior capsules photographed with imagenet^® ^digital photography system and Topcon^® ^camera system at standardised settings. Images were subsequently stored onto disc. Each patient underwent Nd: YAG laser capsulotomy via a set protocol by one surgeon. Patients returned one week later and had further vision testing. They were again dilated and posterior capsules photographed at standardised settings. Full details of the experiment and image acquisition methods are described in a previous publication.[9] Once a new composite image was created, free of external reflections, it was entered into the AA software. The AA program analysed the images created and calculated a score based on an entropy calculation of texture as described above.

Assessment of visual relevance of the analysis system involved seeking evidence for construct validity and convergent validity. Evidence for construct validity was sought by assessing PCO scores of patients with visually significant PCO. These were patients referred for Nd: YAG capsulotomy. The analysis was repeated after YAG capsulotomy when visually significant PCO is in clinical practice significantly reduced. The AA system scores should also be expected to calculate significantly lower values. Paired t-test was used to compare the two groups and results plotted graphically.

For evidence of convergent validity, results of PCO analysis were correlated with vision. Specifically, the difference in PCO quantification by the AA system before and after YAG was compared to improvement in both LogMAR visual acuity and contrast sensitivity for each patient using regression analysis. Graphical representation of results was analysed as well as the indices of regression analysis.

### Testing for reliability

To assess interobserver reliability of the AA system, two trained observers performed analysis on a sample of 20 images. These images included both pre- and post- Nd:;Yag capsulotomy, as well as a selection of images with no PCO. The ICC (intraclass correlation coefficient) was calculated, as well as the coefficient of repeatability and the coefficient of variation. In addition, a Bland-Altman plot to graphically examine the repeatability was plotted.

## Results

### Process and use of developed system

In order to use the system, at present users must have access to Matlab, which is commonplace in scientific and academic institutions. The authors are however currently working on a compiled version that would be usable without any other specialist software. There is free access to the system by contacting the authors.

Once loaded, the AA system asks the user to input an image, and through a menu driven mechanism loads the particular image ready for analysis. The image is converted to greyscale and presented on screen. A cross-hair appears on the image surface and the user clicks on the top left of the image's area of interest and then on the bottom right, thus delineating the region that is to be measured. The experimenter may choose this to be the area within a capsulorhexis, area within a pupil's borders or spreading to the extent of visible intraocular lens, depending on research needs. The program then completes the process of image processing and analysis without further input, and is thus highly objective. The processed images are displayed along with final texture measures.

### Testing for validity

#### Progression of PCO over time

**Figure 3:**
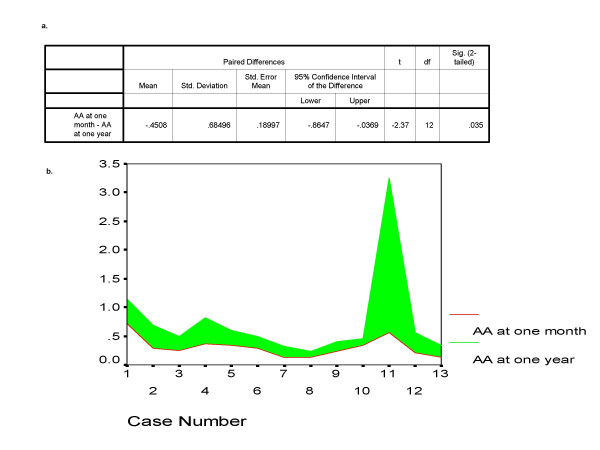
Progression in AA scores between patients one month and 12 months after surgery; a. Paired Samples Test, b. Differences graphically displayed

Analyses of frequency distribution of contrast sensitivity and of PCO scores show distributions that can be considered normal. PCO scores in 12 patients one month after surgery compared to 12–18 months post surgery is demonstrated in fig. 3. Paired T test showed significant differences in the two sets of values (p = 0.035). It is evident that the AA system is able to differentiate between patients with early PCO and no PCO and also to be sensitive to progression of PCO.

#### Visual relevance of the analysis system

##### Construct validity

**Figure 4:**
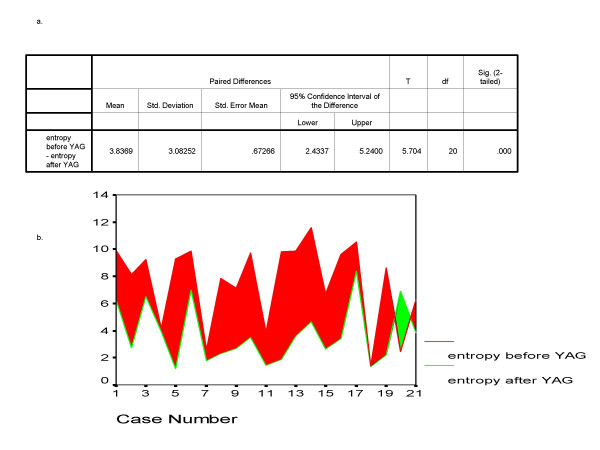
Comparison of AA scores of patients before and after Nd:YAG capsulotomy; a. Statistical analysis, b. Graphically displayed

Analyses of frequency distribution of PCO scores show distributions that can be considered normal. A paired T-test for parametric data was performed for the 21 patients and show a significant difference between AA scores before and after Nd: YAG laser treatment (P < 0.001). Validity evidence by extreme group testing of construct is therefore demonstrated.(fig. 4.)

##### Convergent validity

Linear regression was used in assessing the impact of the change in the AA score on change in visual function measured after Nd: YAG capsulotomy.

**Figure 5:**
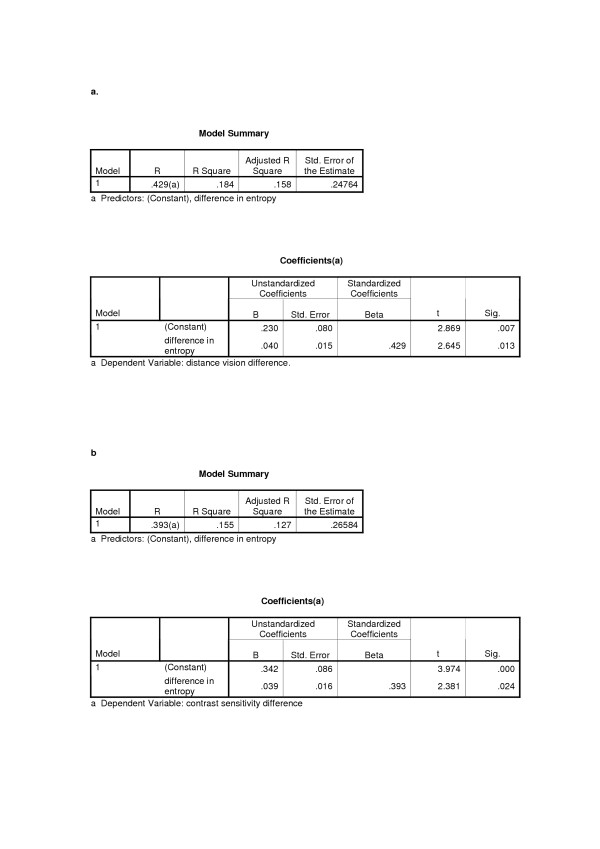
Improvements in vision compared to improvements in AA score, after capsulotomy; a. Regression of distance vision improvement against improvement in AA score, b. Regression analysis of contrast sensitivity improvement against improvement in AA score

The first dependent variable to be studied was improvement in best-corrected distance LogMAR visual acuity (DLVA). Linear regression analysis showed that the standardised coefficient was .43 with significance of 0.013.(fig. 5a) Thus, improvements in distance vision after Nd: YAG capsulotomies are shown to be strongly correlated to the entropy score for PCO in the AA system.

**Figure 6:**
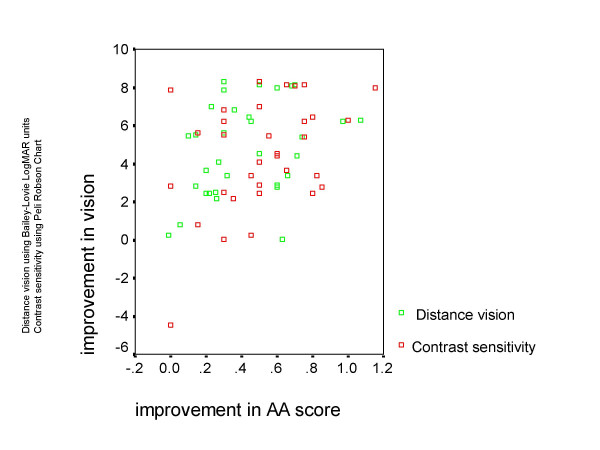
Improvement in contrast sensitivity and distance vision after capsulotomy v improvement in AA scores after capsulotomy

The second dependent variable to be studied was improvement in contrast sensitivity (CS). Linear regression analysis showed that the standardised coefficient was .39 with significance of 0.02.(fig. 5b) Thus, improvements in contrast sensitivity after Nd:YAG capsulotomies are strongly correlated to the differences in entropy scores for PCO in the AA system. A scatter graph demonstrates this correlation of AA score with contrast sensitivity and visual acuity (fig. 6)

### Testing for Reliability

A total of 22 images were analysed once by two observers. The ICC was 0.995 (95% confidence intervals 0.988 to 0.998). Coefficient of Repeatability was 0.559 (ie 95% of repeated measures would be expected to be within this margin).

**Figure 7:**
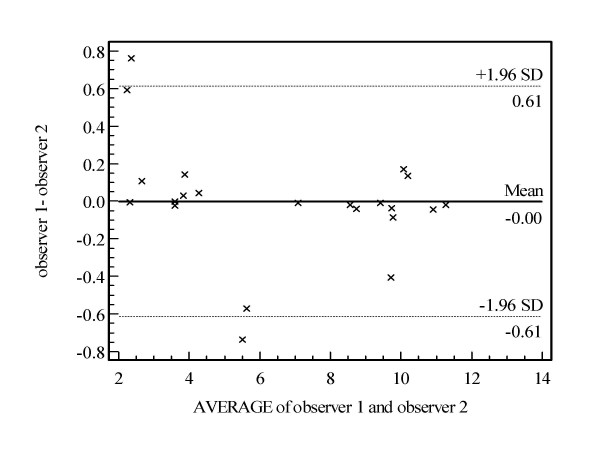
Bland-Altman Plot of interobserver repeatability of AA score between observer 1 and observer 2

A scatter plot (Bland-Altman) showed no relationship between the mean value for each image by the two observers against the standard deviation (fig 7), and we were able to calculate the coefficient of variation, as 3.3% ± 6.6%. These values represent excellent reliability.

## Discussion

This paper describes a logical evidence-based development of a system of analysis of PCO. More importantly, it has presented evidence for the validity and reliability of the AA system. A mass of different textural image analysis tools exist in imaging science [13], but this study has provided evidence that the use of the statistical analysis of the image histogram to calculate entropy can provide valid information on the clinical significance of PCO and on progression of smaller amounts of PCO in clinical trials. Reliability was high, as expected in such a computerised objective system, only limited by variations in actual areas chosen to be examined by the experimenters when operating the program.

The current system of analysis has evolved through the planning, writing, testing and rewriting of many component programming procedures to overcome many practical and theoretical challenges.

On designing the experiments and system of analysis above it was initially considered that various different types of analysis might be required in order to accurately predict different types of visual function loss. However, regression analysis shows that both contrast sensitivity and visual acuity are related to the entropy of the acquired images. The authors suggest that this function calculated by the specific mechanism of the AA system should be used as the outcome measure for the assessment of PCO for clinical and experimental studies.

Evidence has been presented for this system's validity and reliability. Although it is freely available to the scientific community, users should perform their own validity studies incorporating their specific photography systems and procedures for acquiring images and removal of light reflections. At present the AA system requires a Matlab platform to operate, but a compiled version is in progress that would be usable without any prior software. Also, a system for registration based removal of light images is being tested and developed.

## Conclusion

Image analysis with the AA system provides an open access, objective, valid and reliable method of PCO quantification for advancement of research into this common cause for post operative morbidity. We anticipate that with greater use, additional information will lead to even further evidence-based refinements and updates to the system.

## Competing interests

The author(s) declare that they have no competing interests.

## Authors' contributions

Tariq Aslam devised the system of analysis and did all the software programming. Niall Patton collaborated in testing of the system, in particular the reliability studies and reviewed system assessment.

Jim Graham assessed and contributed to the whole paper from an image processing viewpoint.

## Pre-publication history

The pre-publication history for this paper can be accessed here:
